# Influence of viscous dissipation and Joule heating on MHD bio-convection flow over a porous wedge in the presence of nanoparticles and gyrotactic microorganisms

**DOI:** 10.1186/s40064-016-3718-8

**Published:** 2016-11-30

**Authors:** Umar Khan, Naveed Ahmed, Syed Tauseef Mohyud-Din

**Affiliations:** 1 COMSSATS Institute of Information Technology, Abbottabad, Pakistan; 2 Department of Mathematics, Faculty of Sciences, HITEC University, Taxila Cantt, Taxila, Pakistan

**Keywords:** Nanofluids, Joule heating, Viscous dissipation, Gyrotactic microorganisms, Porous wedge, Numerical solution

## Abstract

**Background:**

The flow over a porous wedge, in the presence of viscous dissipation and Joule heating, has been investigated. The wedge is assumed to be saturated with nanofluid containing gyrotactic microorganisms. For the flow, magneto-hydrodynamic effects are also taken into consideration. The problem is formulated by using the passive control model. The partial differential equations, governing the flow, are transformed into a set of ordinary differential equations by employing some suitable similarity transformations.

**Results:**

A numerical scheme, called Runge–Kutta–Fehlberg method, has been used to obtain the local similarity solutions for the system. Variations in the velocity, temperature, concentration and motile micro-organisms density profiles are highlighted with the help of graphs. The expressions for skin friction coefficient, Nusselt number, Sherwood number and motile micro-organisms density number are obtained and plotted accordingly. For the validity of the obtained results, a comparison with already existing results (special cases) is also presented.

**Conclusion:**

The magnetic field increases the velocity of the fluid. Injection at the walls can be used to reduce the velocity boundary layer thickness. Thermal boundary layer thickness can be reduced by using the magnetic field and the suction at the wall. The motile microorganisms density profile is an increasing function of the bioconvection Pecket number and bioconvection constant. The same is a decreasing function of *m*, *M* and *Le*. The skin friction coefficient increases with increasing *m* and $M$. Nusselt number and the density number of motile microorganisms are higher for the case of suction as compared to the injection case. The density number of motile microorganisms is an increasing function for all the involved parameters.

## Background

In recent times, scientists and researchers are keenly working on the ways to improve the heat transfer characteristics of the fluids used in everyday life. For this purpose, many theoretical as well as practical studies have been presented over the years. Choi (1995), in one of the important studies in this regard, presented a useful and important model. The proposed model uses nanoparticles to improve the heat transfer characteristics of the fluids like water, kerosene and the other traditional fluids. He proved that the thermal properties of these fluids (termed as base fluids) can be enhanced by the addition of nano particles (Choi et al. 2001). After this benchmark study, many researchers dedicated their time to work in the field of nanofluids. In another study, Buongiorno (2006), suggested a model that incorporates the Brownian motion and thermophoresis effects in energy and concentration equations. Working on the idea of Buongiorno, Khan and Pop (2010) studied the boundary layer flow of nanofluid over a stretching surface. Makinde and Aziz (2011) extended the same idea for the case of convective boundary conditions. Several studies on this topic have been presented over the years. Some of the most relevant and useful ones can be seen in (Sheikholeslami and Ellahi 2015a, b; Ellahi et al. 2015; Khan et al. 2015; Mohyud-Din et al. 2015a, b; Gul et al. 2015a, b) and the references therein.

Flow over a wedge has gained interest of many researchers due to the practical applications it has in polymer processes, cooling or heating of films/sheets, insulating materials, conveyor belts, cylinders and metallic plates. The seminal work regarding the flow over a wedge has been carried out by Falkner and Skan (1931). Their study considers a fixed wedge and the absence of any external forces. Hartree (1937) and Koh and Hartnett (1961), extended the idea of Falkner and Skan by considering the various factors involved, and, provided an extended solution to the traditional wedge problem. Suction/injection and variable wall temperature were the major factors considered by them. Magneto-hydrodynamic effects in the flow over a wedge were considered by Thakar and Pop (1984). Khan and Pop (2013) presented the boundary layer flow past a wedge moving in a nanofluid. Khan et al. (2015) used the Xue model to analyze the flow of carbon nanotubes suspended nanofluid over a static/moving wedge. Khan et al. (2015) used the nonlinear form of thermal radiation to study the flow properties in a porous wedge under the influence of magnetic field.

Bioconvection is due to the macroscopic convective motion of fluid caused by the density gradient. The collective swimming of the motile microorganisms creates the bioconvection. The swimming of these self-propelled motile microorganisms results in increased values for the density that cause bioconvection. Studies related to bioconvection can be seen in Kuznetsov (2010), Khan and Makinde (2014), Nield and Kuznetsov (2006), Avramenko and Kuznetsov (2004), Makinde and Animasaun (2016a, b), Mutuku and Makinde (2014), Khan et al. (2014) and the references therein. All these researchers considered the flow by taking nanofluids and concluded that motion due to self-propelled microorganisms result in enhancement in mixing and thus preventing nanoparticle cluster.

A careful literature survey reveals that to date, no study is available which considers the boundary layer flow of a nanofluid over a wedge in presence of microorganisms. To fill up this gap, we present here a mathematical study analyzing the flow of a nanofluid over a porous wedge in the presence of gyrotactic microorganisms. MHD along with the Joule heating effects for the flow are also taken into consideration. The flow analysis is carried out after reducing the equations governing the flow into a set of ordinary differential equations. The solution of the problem is obtained numerically. The graphs are plotted to highlight the effects of various emerging parameters. A comprehensive discussion over those graphs is also presented.

## Problem formulation

**Fig. 1:**
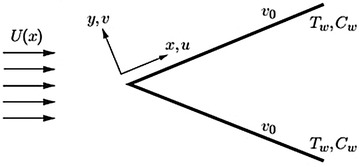
Schematic diagram of the flow problem

Consider the boundary layer flow past a stretchable wedge. The wedge is assumed to be moving with a velocity *u*_*w*_(*x*) in a water-based nanofluid saturated by gyrotactic microorganisms. The free stream velocity is taken to be *u*_*e*_(*x*). Further, there is no nanoparticle agglomeration, the effect of nanoparticles on the swimming direction of microorganisms and on the velocity of swimming of microorganisms. The assumption to be valid, we assume that the suspension of nanoparticles is dilute. To formulate the flow phenomena, we have considered a Cartesian coordinate system. The coordinates along the surface and normal to it, are denoted by *x* and *y*, respectively (see Fig. 1). A uniform magnetic field is applied parallel to the y-axis. The induced magnetic field is assumed to be negligible. The viscous dissipation and joule heating effects are also taken into consideration while modeling the energy equation. At the surface of the wedge, a constant suction or injection is imposed. Under the aforesaid assumptions, and using the scale analysis of Buongiorno (2006) and Kuznetsov (2010), the boundary layer equations governing the flow can be written as follows:

1 $$ \frac{{\partial\check{u}}}{{\partial\check{x}}} + \frac{{\partial\check{v}}}{{\partial\check{y}}} = 0, $$

2 $$ \check{u} \frac{{\partial \check{u} }}{{\partial \check{x} }} + \check{v} \frac{{\partial \check{u} }}{{\partial \check{y} }} = u_{e} \frac{{du_{e} }}{{d\check{x} }} + \upsilon \frac{{\partial^{2} \check{u} }}{{\partial \check{y}^{2} }} - \frac{{\sigma B_{0}^{2} }}{\rho }\left( {\check{u} - u_{e} } \right), $$

3 $$ \check{u} \frac{{\partial \check{T} }}{{\partial \check{x} }} + v\frac{{\partial \check{T} }}{{\partial \check{y} }} = \alpha \frac{{\partial^{2} \check{T} }}{{\partial \check{y}^{2} }} + \tau \left[ {D_{B} \frac{{\partial \check{C} }}{{\partial \check{y} }}\frac{{\partial \check{T} }}{{\partial \check{y} }} + \left( {\frac{{D_{T} }}{{T_{\infty } }}} \right)\left( {\frac{{\partial \check{T} }}{{\partial \check{y} }}} \right)^{2} } \right] + \frac{\mu }{{\left( {\rho C_{p} } \right)_{f} }}\left( {\frac{{\partial \check{u} }}{{\partial \check{y} }}} \right)^{2} + \frac{{\sigma B_{0}^{2} }}{{\left( {\rho C_{p} } \right)_{f} }}\left( {u - u_{e} } \right) ^{2} , $$

4 $$ \check{u} \frac{{\partial \check{C} }}{{\partial \check{x} }} + \check{v} \frac{{\partial \check{C} }}{{\partial \check{y} }} = D_{B} \frac{{\partial^{2} \check{C} }}{{\partial \check{y}^{2} }} + \left( {\frac{{D_{T} }}{{T_{\infty } }}} \right)\frac{{\partial^{2} \check{T} }}{{\partial \check{y}^{2} }}, $$

5 $$ \check{u} \frac{{\partial \check{n} }}{{\partial \check{x} }} + \check{v} \frac{{\partial \check{n} }}{{\partial \check{y} }}+ \frac{{\partial (\check{n}\overbrace{v})}} {{\partial \check{y} }} = D_{n} \frac{{\partial^{2} \check{n} }}{{\partial \check{y}^{2} }}.$$

In above equations, $\check{u}$ and $\check{v}$ are the components of velocity in $\check{x}$ and $\check{y}$ directions, respectively. $\check{T}$, is the temperature of the fluid, *ρ*is the density of nanofluid, $\mu$ is the viscosity of nanofluid and $\alpha$ is the thermal diffusivity of nanofluid. Moreover, $\tau = \frac{{\left( {\rho C} \right)_{p} }}{{\left( {\rho C} \right)_{f} }} ;$ where *C* is the volumetric expansion coefficient and $\rho_{p}$ the density of the particles. Furthermore, $\check{n}$ is the density of the motile microorganisms, $\overbrace{v}=\left( {\frac{(bWc)}{\Delta C}}\right)\nabla C$, is the velocity vector representing the cell swimming in nanofluids, *Dn* is the diffusivity of microorganisms, *b* is the chemotaxis constant [m] and *Wc* is the maximum cell swimming speed [m/s].

The boundary conditions for the problem are:

$$ \check{u} = u_{w} \left( x \right),\quad\check{v} = v_{0} ,\quad\check{T} = T_{w} , \quad D_{B} \frac{{d\check{C} }}{{d\check{y} }} + \frac{{D_{T} }}{{T_{\infty } }} \frac{{d\check{T} }}{{d\check{y} }} = 0,\quad \check{n} = n_{w},\quad {\text{at}}\;\check{y} = 0, $$

6 $$ \check{u} = u_{e} \left( x \right),\quad   \check{T} = T_{\infty },\quad   C = C_{\infty },\quad  \check{n} = n_{\infty }  \quad{\text{as}}\;y \to \infty , $$

For a mathematical analysis of the problem, we assume that *u*_*w*_(*x*) and *u*_*e*_(*x*) have the following form:

$$ u_{w} \left( x \right) = ax^{m} ,\quad u_{e} \left( x \right) = cx^{m} , $$

where, *a* and *c* are positive constants; besides, $m = \frac{\beta }{{\left( {2 - \beta } \right)}}\left( {0 \le m \le 1} \right) \cdot \beta$ here is Hartee pressure gradient parameter which corresponds to *β* = *Ω*/2 for a total wedge angle Ω.

We seek a similarity solution for the Eqs. (1)–(4) of the form,

7 $$ \psi = \left( {\frac{{2U_{e} x\nu }}{1 + m}} \right)^{{\frac{1}{2}}} F\left( \eta \right),\quad \theta \left( \eta \right) = \frac{{T - T_{\infty } }}{{T_{w} - T_{\infty } }},\quad \phi \left( \eta \right) = \frac{{\check{C} - C_{\infty } }}{{C_{\infty } }},\quad \eta = \left( {\frac{{\left( {1 + m} \right)U_{e} }}{2x\nu }} \right)^{{\frac{1}{2}}} y. $$

$\psi$ in Eq. (7) is the stream function that can be defined in a usual way. Besides, $u = \frac{\partial \psi }{\partial x}$ and $v = - \frac{\partial \psi }{\partial y}$. Using Eq. (5) and the stream function into Eqs. (1)–(4), we get the following system of nonlinear differential equations,

8 $$ F^{{{\prime \prime \prime }}} + FF^{{\prime \prime }} + \left( {\frac{2m}{m + 1}} \right)\left( {1 - \left( {F^{{\prime }} } \right)^{2} } \right) + M^{2} \left( {1 - \left( {F^{{\prime }} } \right)} \right) = 0, $$

9 $$ \theta^{{\prime \prime }} + \Pr f\theta^{{\prime }} + \Pr Nb\phi^{{\prime }} \theta^{{\prime }} + \Pr Nt\theta^{{{\prime }2}} + \Pr Ecf^{{{\prime \prime }2}} + \Pr EcM^{2} \left( {f^{{\prime }} - 1} \right)^{2} = 0, $$

10 $$ \phi^{{\prime \prime }} + LePrf\phi^{{\prime }} + \frac{Nt}{Nb}\theta^{{\prime \prime }} = 0. $$

11 $$ \chi^{{\prime \prime }} + PrLb\left( {f\chi^{{\prime }} } \right) - Pe\left( {\phi^{{\prime }} \chi^{{\prime }} + \phi^{{\prime \prime }} \left( {\sigma + \chi } \right)} \right) = 0. $$

The boundary conditions also get transformed to

12 $$ F\left( 0 \right) = \frac{2}{m + 1}S,\quad F^{{\prime }} \left( 0 \right) = 0,\quad  \theta \left( 0 \right) = 1,\quad Nb\phi^{{\prime }} \left( 0 \right) + Nt\theta^{{\prime }} \left( 0 \right) = 0,\quad  \chi \left( 0 \right) = 1 $$

13 $$ F^{{\prime }} \left( \infty \right) = 1,\quad \theta \left( \infty \right) = 0,\quad  \phi \left( \infty \right) = 0, \quad \chi \left( \infty \right) = 0. $$

In the above equations, *m* is the pressure gradient parameter and *S* is the suction/injection parameter. *S* > 0 shows that there is injection at the wall while *S* < 0 corresponds to the cases involving suction at the wall. $M = \frac{{2\sigma B_{0}^{2} }}{{\rho \left( {1 + m} \right)U_{e} \left( x \right)}}$, $Ec = \frac{{u_{w}^{2} }}{{\left( C \right)_{f} \left( {T_{w} - T_{\infty } } \right)}},$ $Pr = \frac{\nu }{\alpha }$, $Le = \frac{\alpha }{{D_{B} }}$, $Nb = \frac{{\left( {\rho C} \right)_{p} D_{B} C_{\infty } }}{{\left( {\rho C} \right)_{f} \nu }}$, $Nt = \frac{{\left( {\rho C} \right)_{p} D_{T} \left( {T_{w} - T_{\infty } } \right)}}{{\left( {\rho C} \right)_{f} T_{\infty } \nu }}$, $Lb = \frac{\alpha }{Dn}, \quad Pe = \frac{bWc}{Dm}$ and $\sigma = \frac{{n_{\infty } }}{{\Delta n_{w} }}.$ represent magnetic number, Prandtl number, Lewis number, Brownian motion parameter, thermophoresis parameter, bioconvection Lewis number, the bioconvection Pecket number and the bioconvection constant, respectively.

Some physical quantities of interest are the skin friction coefficient and Nusselt number. They are defined respectively as:

$$ Re_{x}^{{\frac{1}{2}}} C_{f} = \sqrt {\frac{m + 1}{2}} F^{{\prime \prime }} \left( 0 \right), $$

and

$$ Re_{x}^{ - 1/2} Nu = \sqrt {\frac{m + 1}{2}} \theta^{{\prime }} \left( 0 \right). $$

It is pertinent to mention here that the Sherwood number for the passive control model becomes identically zero, i.e.

$$ Re_{x}^{ - 1/2} Sh = \sqrt {\frac{m + 1}{2}} \phi^{{\prime }} \left( 0 \right) = 0, $$

The microorganism density number in dimensionless form will reduce to:

$$ Re_{x}^{ - 1/2} Nn = \sqrt {\frac{m + 1}{2}} \chi^{{\prime }} \left( 0 \right). $$

Here, $Re_{x} = \frac{{u_{e} x}}{\nu }$ is the local Reynold number.

## Methods

The system of governing equations at hand is solved by employing a reputable numerical scheme known as Runge–Kutta–Fehlberg (RKF) Method, combined with the shooting technique. To obtain the local similarity solutions of the system, the shooting method converts a system of boundary value problems to a corresponding system of initial value problems. The resulting system can readily be solved by RKF method. The step size is taken as 0.001 and the convergence criteria is taken as 10^−6^. The asymptotic boundary conditions in Eq. (12) are replaced (by using a fixed value for the similarity variable $\eta_{max}$) as follows:

$$ \eta_{max} = 12, \quad F^{{\prime }} \left( {12} \right) = 1,\quad \theta \left( {12} \right) = 0,\quad \phi \left( {12} \right) = 0. $$

## Results and discussion

**Fig. 2:**
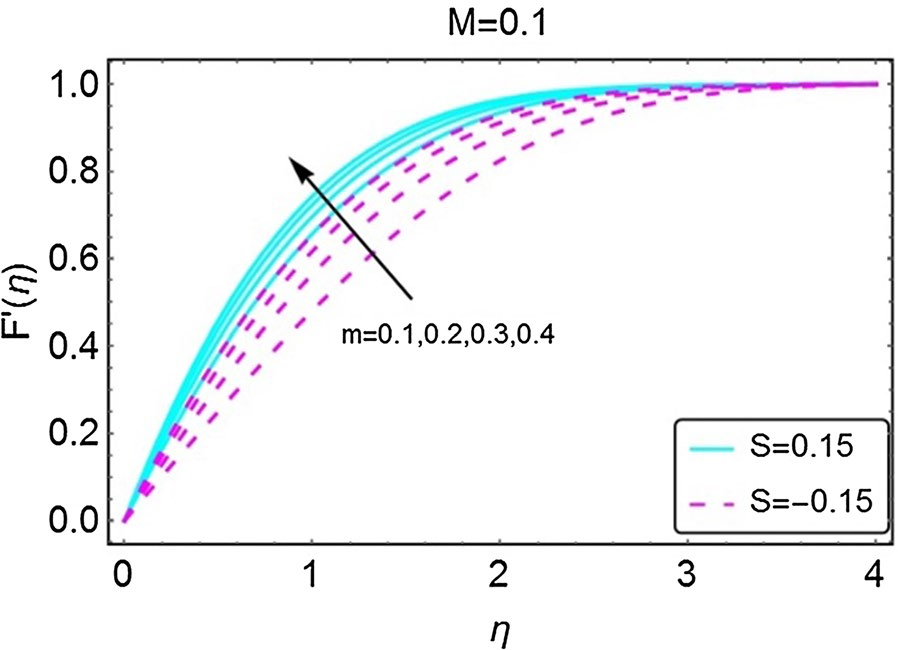
Variation in velocity with increasing values of *m*

**Fig. 3:**
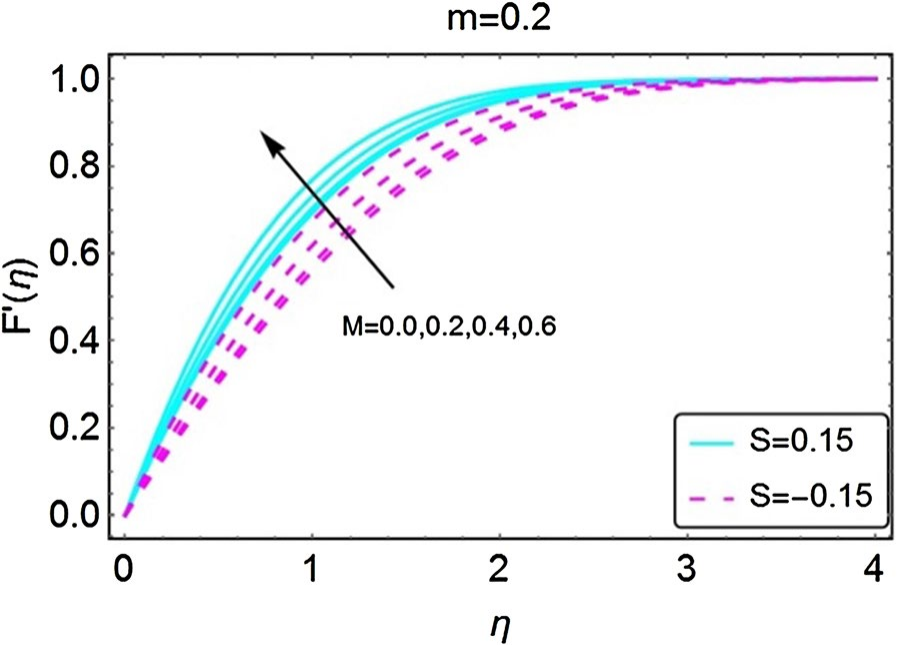
Variation in velocity with increasing values of *M*

In this section, we highlight the influence of some relevant parameters on the velocity, temperature, concentration and motile microorganisms density profiles with the help of graphs. Here, it is pertinent to mention that the solid lines in the graphs represent the case of suction at the wall, while, the dotted lines are for the injection case unless stated otherwise. The variation in velocity for the increasing values of *m* has been given in Fig. 2. A rise in the velocity is clearly evident. It can also be observed that the suction at the wall allows the fluid to enter the plate, that in return increases the velocity of the fluid. The influence of increasing values of magnetic parameter $M$ on the velocity profile is given in Fig. 3. The velocity profile is found to be an increasing function of *M*. A decrease in the boundary layer thickness is seen for increasing values of *M*; as a result, the velocity of the fluid becomes normalized. A higher velocity is observed when there is suction at the wall.

**Fig. 4:**
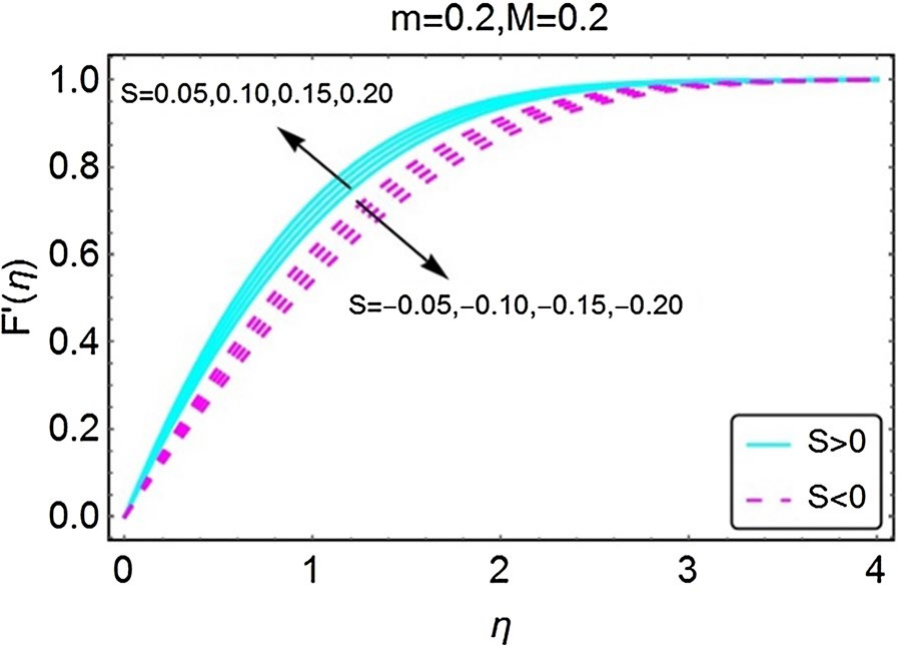
Variation in velocity with variations in *S*

The variations in velocity caused by the suction/injection parameter are depicted in Fig. 4. An increase in injection at the wall gives a rise in the velocity of the fluid. Due to inward movement of the fluid due to injection, more fluid enters the region of the wedge that in return influences the velocity of the fluid quite significantly. The suction of fluid at the wall behaves quite oppositely. The outward movement of the fluid due to suction results in lower concentration fluid inside the wedge region that corresponds to a drop in velocity of the fluid.

**Fig. 5:**
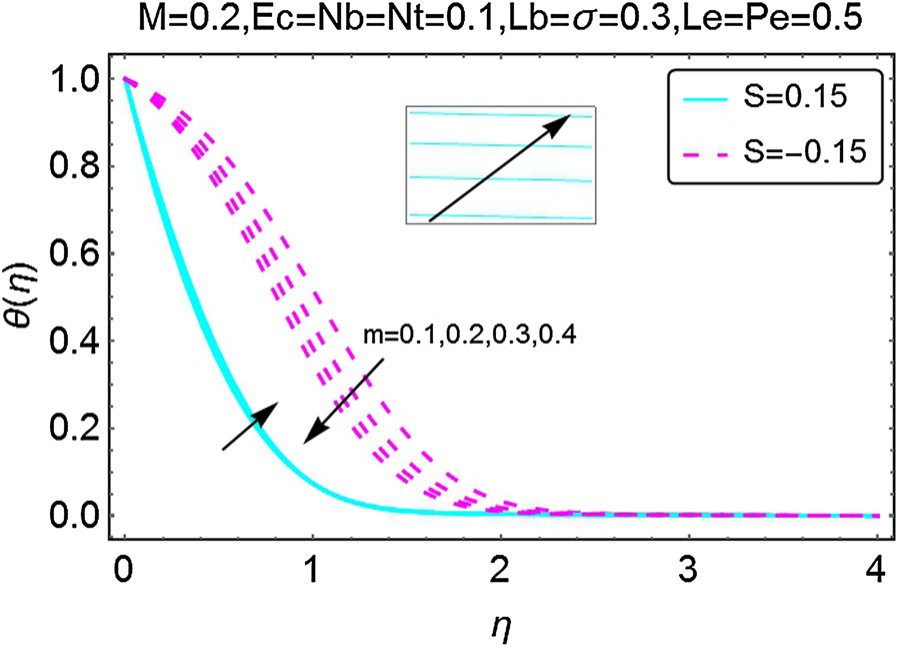
Variation in temperature with variations in *m*

**Fig. 6:**
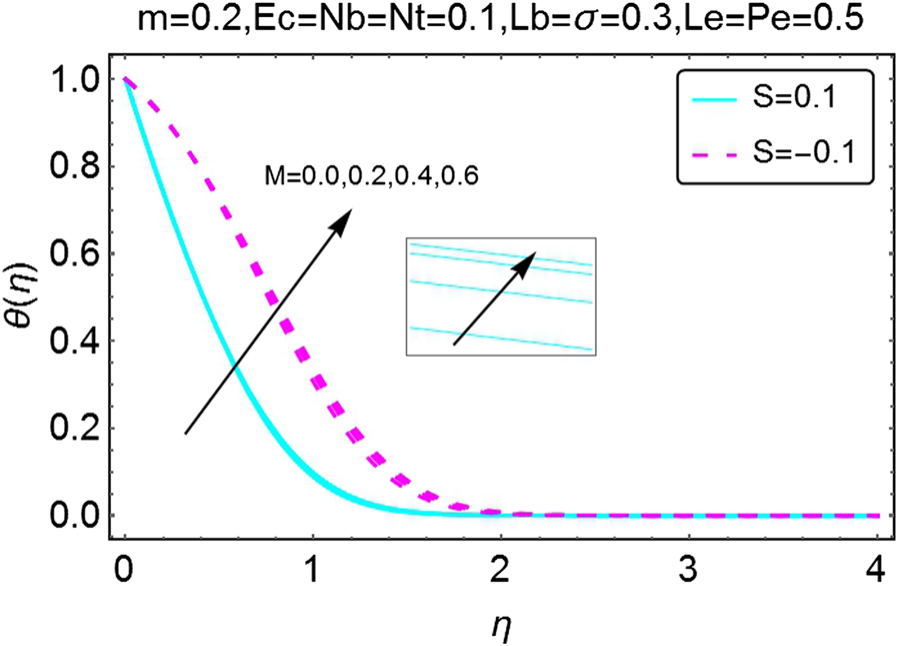
Variation in temperature with variations in *M*

The next set of figures describes the variations in the temperature profile with increasing values of the parameters involved. The value of Prandtl number *Pr* is taken to be 6.2 throughout the manuscript and it corresponds to water. The increment in pressure parameter *m* gives a rise to temperature for the case when there is suction at the wall. For the injection case, the phenomenon is reversed and a decrement in the temperature of the fluid is seen with the increasing values of $m$. The thermal boundary layer also reduces with an increase in *m* for injection case. Due to the presence of Joule heating, stronger the magnetic field higher the temperature of the fluid is. Besides, for both suction and the injection cases, the change in temperature is almost similar (Figs. 5, 6).

**Fig. 7:**
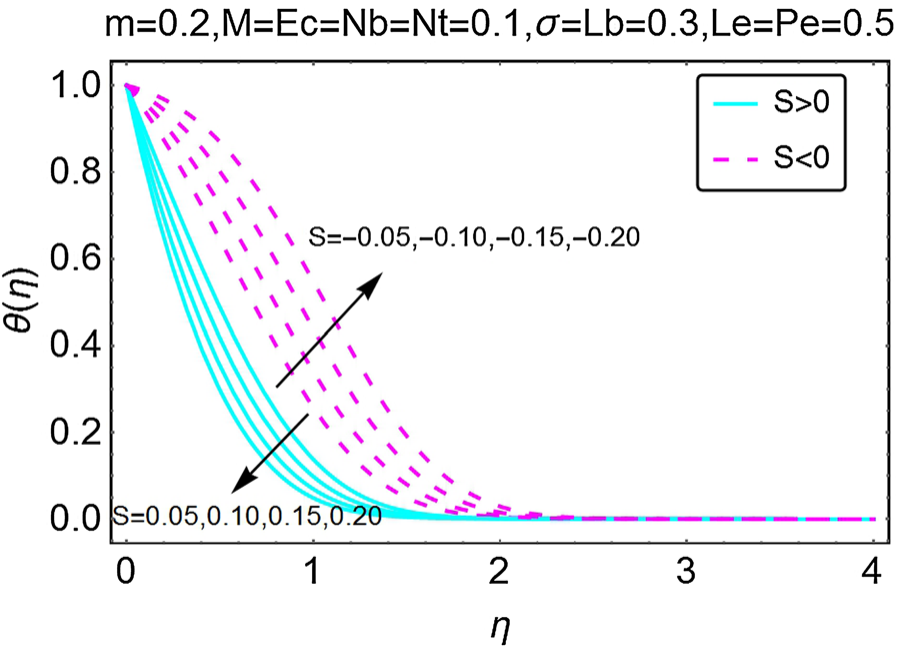
Variation in temperature with variations in *S*

**Fig. 8:**
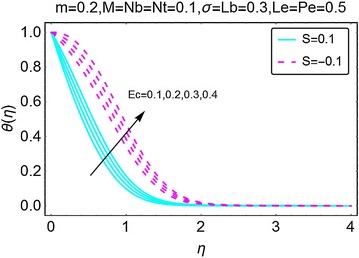
Variation in temperature with variations in *Ec*

The variations in temperature profile for different values of suction/injection parameter $S$ and the viscous dissipation parameter *Ec* are highlighted in Figs. 7 and 8, respectively. From Fig. 7, it can be comprehended that the suction at the wall decreases the temperature profile and also reduces the corresponding boundary layer. This is because of the fact that due to injection, more fluid is dragged out that in return reduces the fluid inside the wedge causing a fall in temperature of the fluid. Suction at the wall gets more fluid inside the wedge and raises the temperature profile and the corresponding boundary layer as seen in Fig. 7. In Fig. 8, the variations in temperature profile with the varying values of Eckert number $Ec$ are highlighted. An upsurge in the temperature of the fluid is seen with increasing $Ec$. Eckert number is due to the presence of velocity term in the energy equation and thus gives a prominent rise in temperature. It is pertinent to mention here that for all the cases involved, the temperature is on a higher side when there is injection at the wall, this is possible physically because due to suction.

**Fig. 9:**
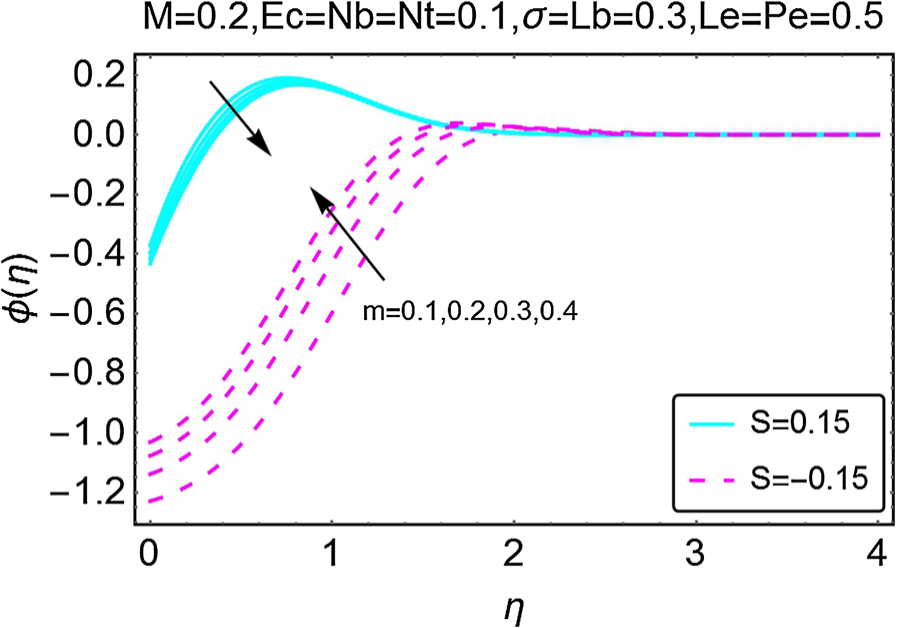
Variation in concentration with variations in *m*

**Fig. 10:**
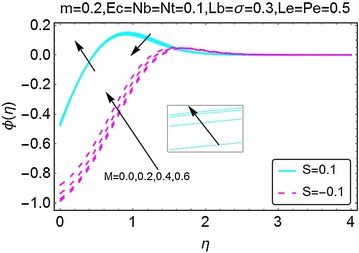
Variation in concentration with variations in *M*

The next set of figures describes the variations in the concentration profile under the influence of various involved parameters. Figure 9 is plotted to highlight the deviations in concentration profile with the increment of $m$. It is noted that when there is suction at the wall, the concentration drops initially; however, after a certain point the concentration starts rising and eventually it gets stable away from the wall of the wedge. With an increment in $m$, the concentration profile varies inversely. For the suction case, an up rise in concentration of the fluid is witnessed at the start of the wedge. Moreover, the profile stabilizes far away from the wedge. Higher concentration for the case of injection as compared to the injection at the wall. The way in which the magnetic parameter $M$ affects the concentration profile is depicted in Fig. 10. A rise in the concentration of the fluid is seen for both suction and injection cases; however, the concentration for the suction case remains on a higher side.

**Fig. 11:**
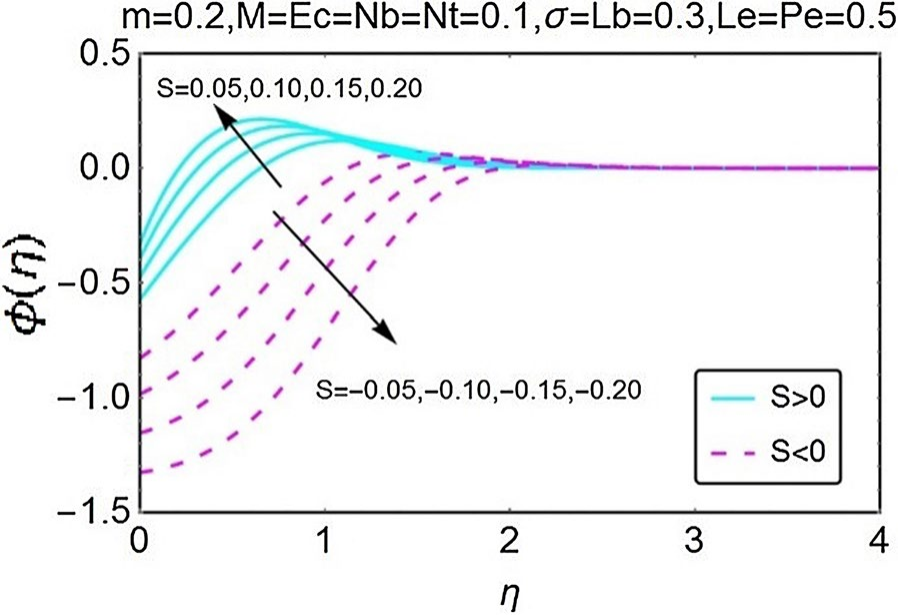
Variation in concentration with variations in *S*

**Fig. 12:**
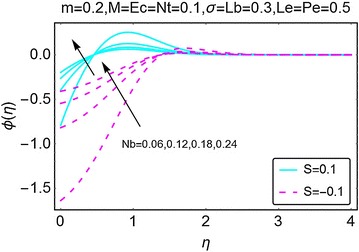
Variation in concentration with variations in *Nb*

Figure 11 displays the variation in concentration with increasing suction/injection at the wall. As suction at the wall increases, the concentration curves lift initially and then they start bending down before stabilizing away from the wall of the wedge. A drop in concentration is seen for the case when the suction at the wall increases. Furthermore, the case of injection has high concentration values as compared to the suction case. In Fig. 12, the behavior of concentration profile with increasing values of Brownian motion parameter *Nb* is portrayed. For the suction case, initially, the concentration increases with a rise in $Nb$ near the wall; however, as we move away from the wall, the concentration suddenly starts decreasing and becomes stable far away from the wall of the wedge. This interesting behavior is due to the presence of passive boundary condition for the concentration profile at the wall of the wedge. For the case of injection at wall, the concentration is seen to be increasing near the wall and it stabilizes far away from it.

**Fig. 13:**
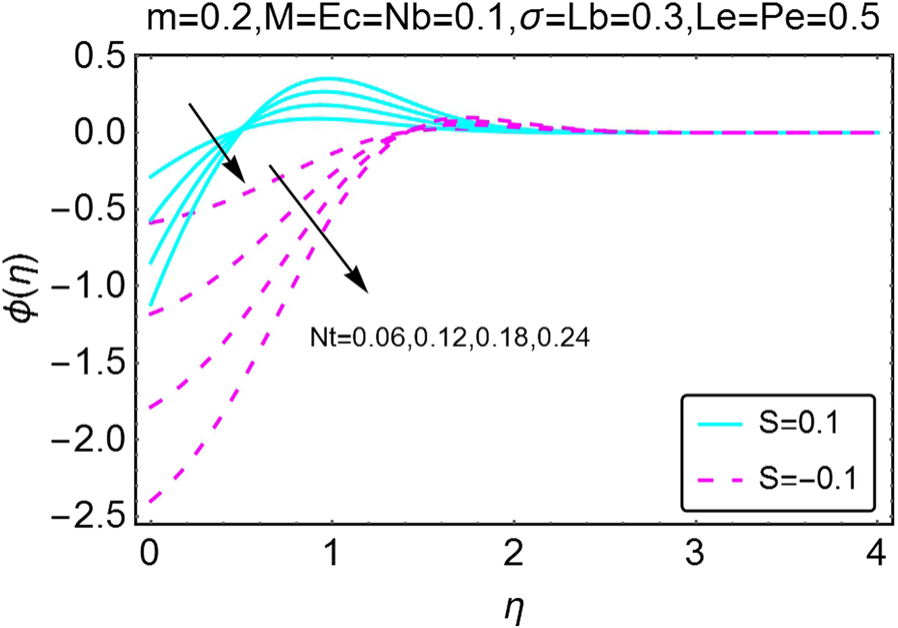
Variation in concentration with variations in *Nt*

**Fig. 14:**
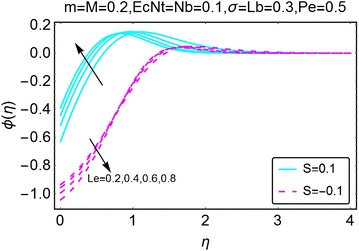
Variation in concentration with variations in *Le*

The behavior of concentration profile under the influence of increasing thermophoresis parameter $Nt$ is highlighted in Fig. 13. When there is injection at the wall, with an increase in $Nt$, the concentration of the fluid decreases near the wall of the wedge and starts increasing slightly away from the wall and stabilizes far away at the end points from the wall of the wedge. For the case of suction at the wall, the behavior is somewhat smooth. Near the wall of the wedge, the concentration decreases with an increase in $Nt$ while it stays uniform far away from the wall. The concentration changes due to the varying values of Lewis number $Le$ are plotted in Fig. 14. When there is injection at the wall, the concentration near the wall decreases. A slightly away from the wall, the behavior is opposite and a rise in concentration is observed with increasing Le. Eventually the concentration becomes stable far away from the wall of the wedge.

**Fig. 15:**
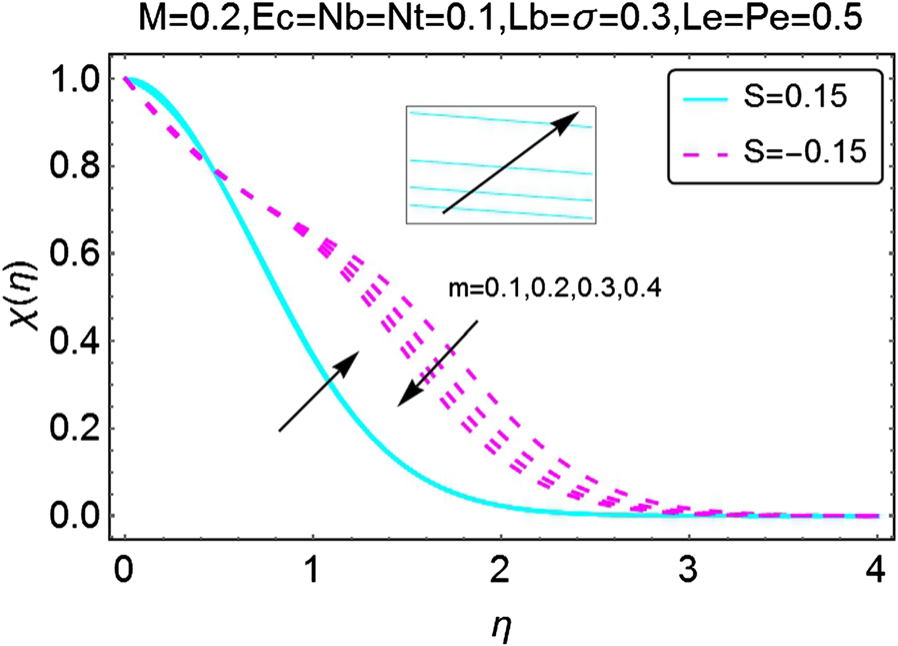
Variation in density of motile microorganisms with variations in *m*

**Fig. 16:**
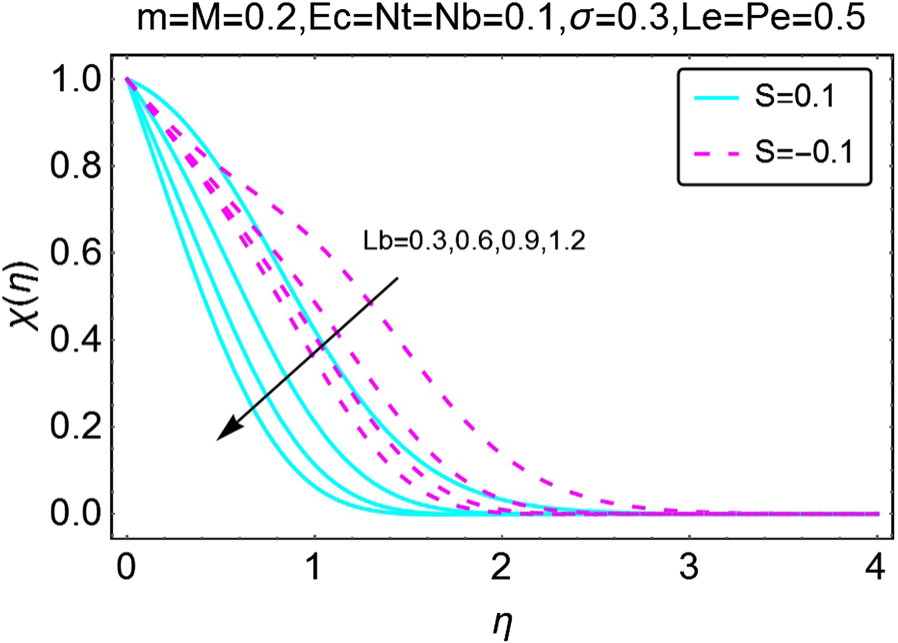
Variation in density of motile microorganisms with variations in *Lb*

**Fig. 17:**
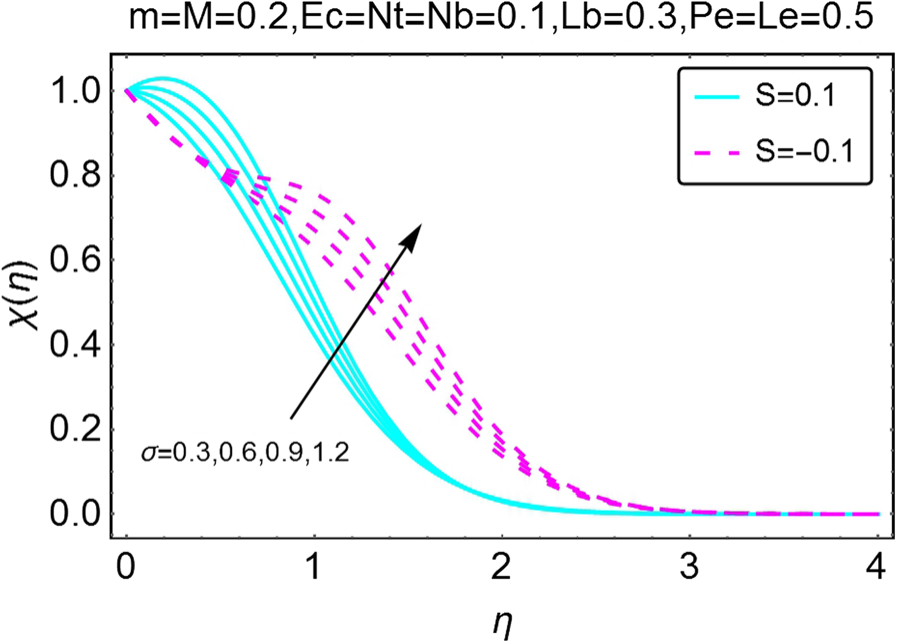
Variation in density of motile microorganisms with variations in *σ*

**Fig. 18:**
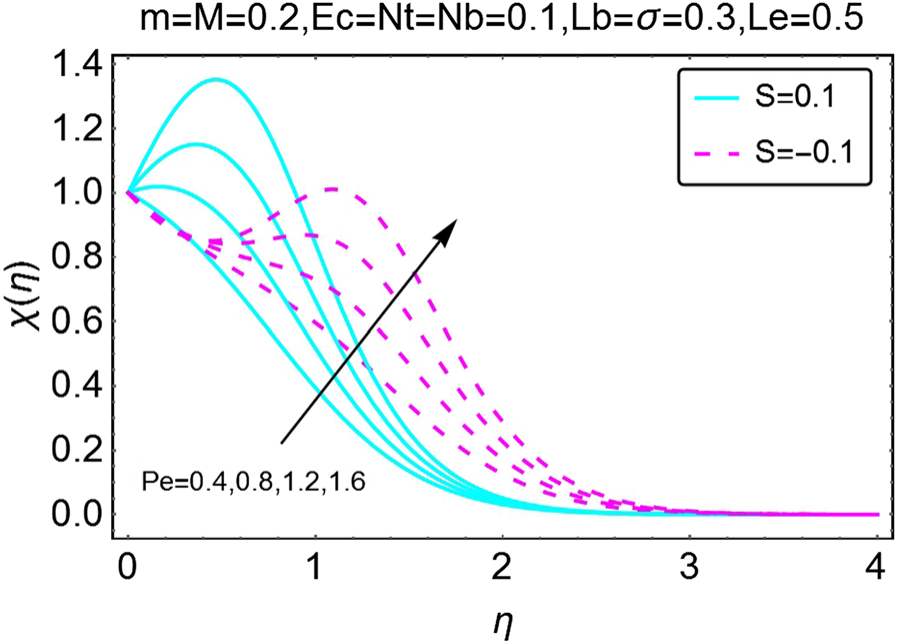
Variation in density of motile microorganisms with variations in $\varvec{Pe}$

The graphical description of the effects of relevant parameters on the motile microorganisms density profile is presented in Figs. 15, 16, 17, and 18. Figure 15 shows the variations in density of motile microorganisms with the increasing values of $m$. It can be seen when there is suction at the wall, the motile microorganisms profile decreases with an increase in $m$. On the other hand, in injection case, the behavior is reversed and the motile microorganisms density profile varies directly with increasing *m*. The corresponding boundary layer thickness is also a decreasing function of increasing $m$. Figure 16 portrays the influence of increasing bio-convection Lewis parameter $Lb$ on the motile microorganisms density profile. A drop in density, and the associated boundary layer thickness, is seen with an increase in $Lb$. Figure 17 describes the effects of increasing bio-convection constant $\sigma$ on the motile microorganisms density profile. An increase in $\sigma$ raises the density profile for both suction and injection cases. Higher values of the density profile are seen for the case of suction. Besides, the associated boundary layer thickness is also an increasing function of increasing $\sigma .$ Fig. 18 displays the effects of bio-convection Pecket number $Pe$ on the microorganisms density profile. An increment in *Pe* increase the density of motile microorganisms density as well as the associated boundary layer thickness. The increment is more prominent in the case of suction as compared to the case of injection. It is worth nothing that for all the cases, density of motile microorganisms is on a higher side when there is injection at the wall of the wedge.

**Fig. 19:**
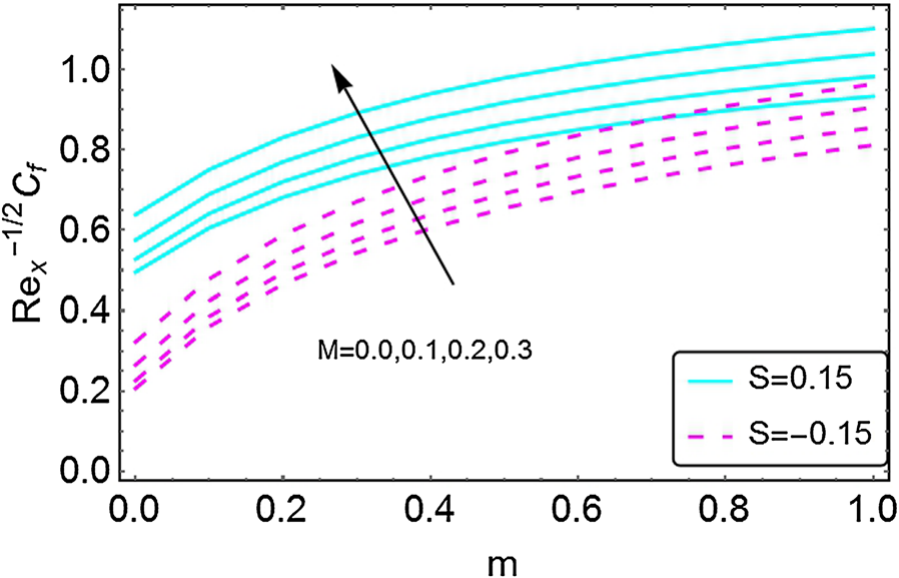
Variation in skin friction coefficient with m and $\varvec{M}$

**Fig. 20:**
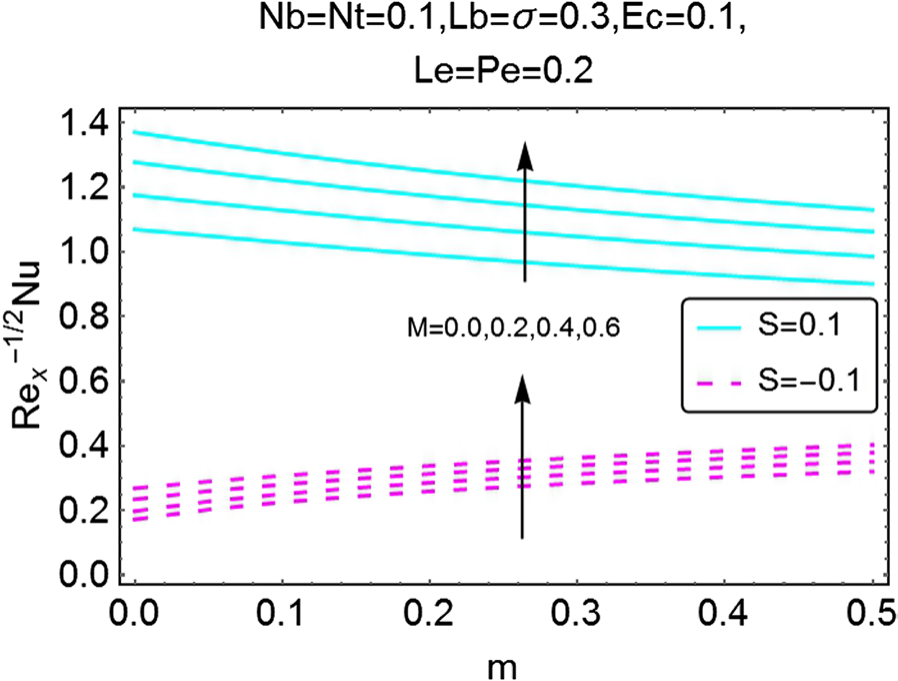
Variation in Nusselt number with *m* and *M*

**Fig. 21:**
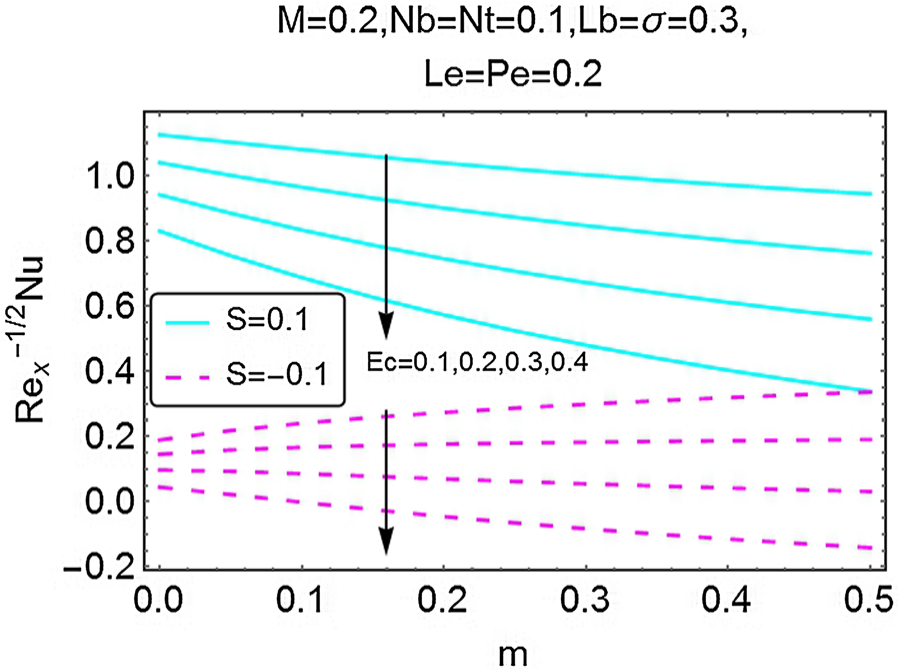
Variation in Nusselt number with *m* and *Ec*

**Fig. 22:**
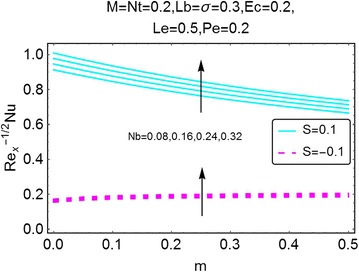
Variation in Nusselt number with *m* and *Nb*

**Fig. 23:**
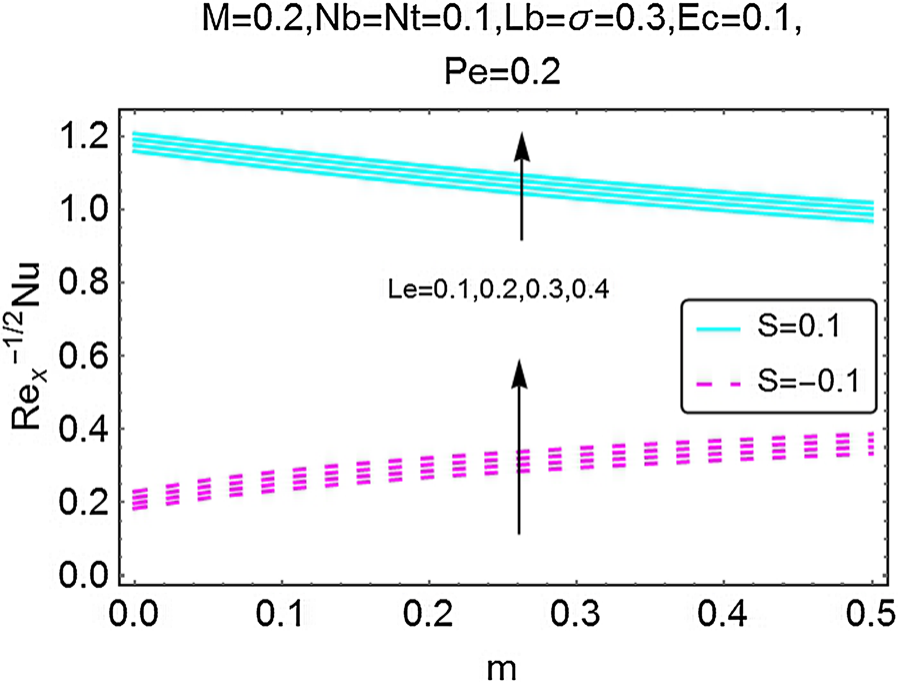
Variation in Nusselt number with *m* and *Le*

**Fig. 24:**
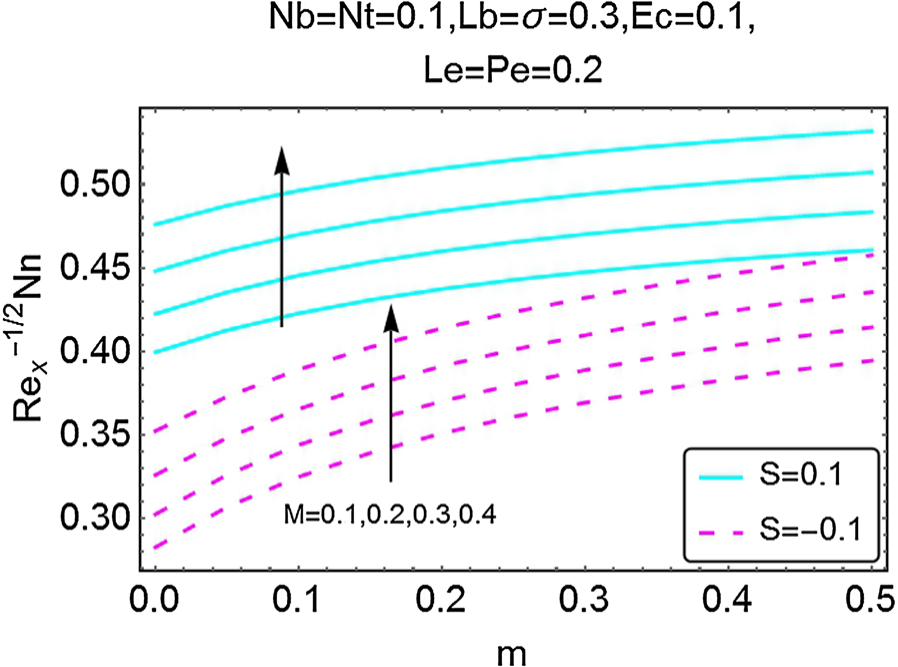
Variation in density number of motile microorganisms with *m* and *M*

**Fig. 25:**
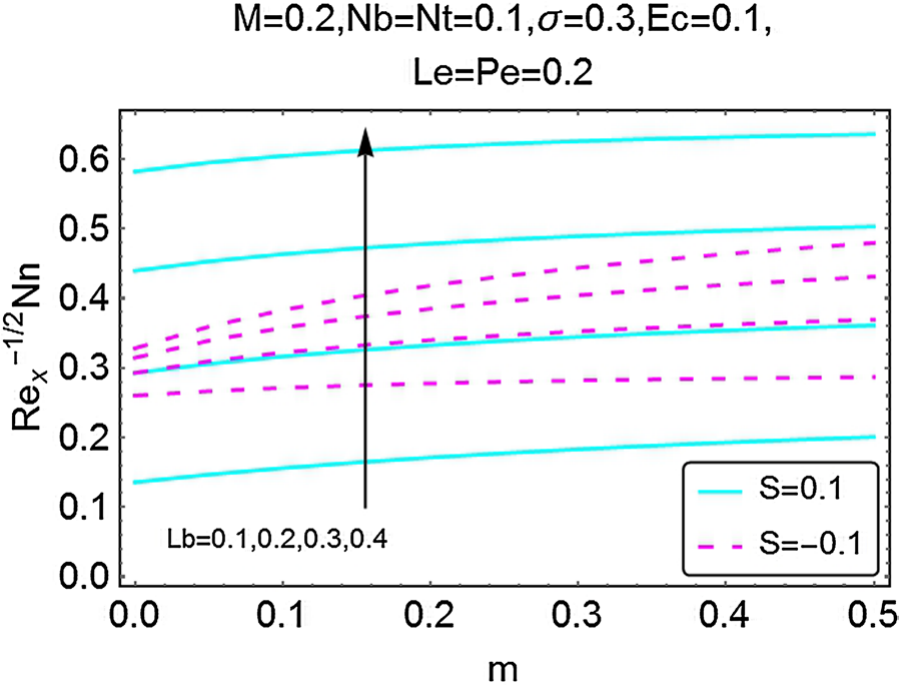
Variation in density number of motile microorganisms with *m* and *Lb*

**Fig. 26:**
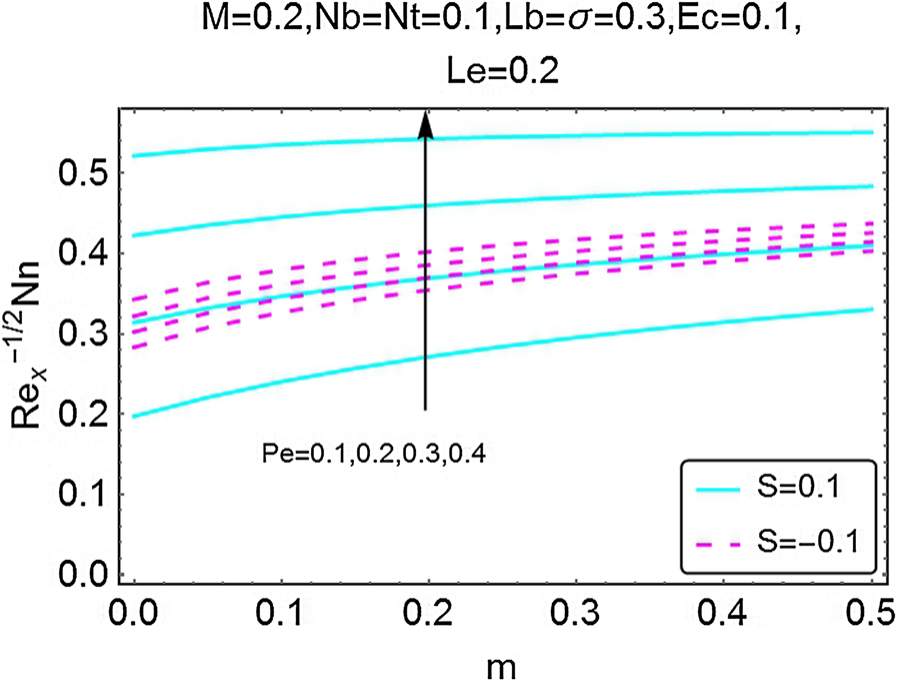
Variation in density number of motile microorganisms with *m* and *Pe*

**Fig. 27:**
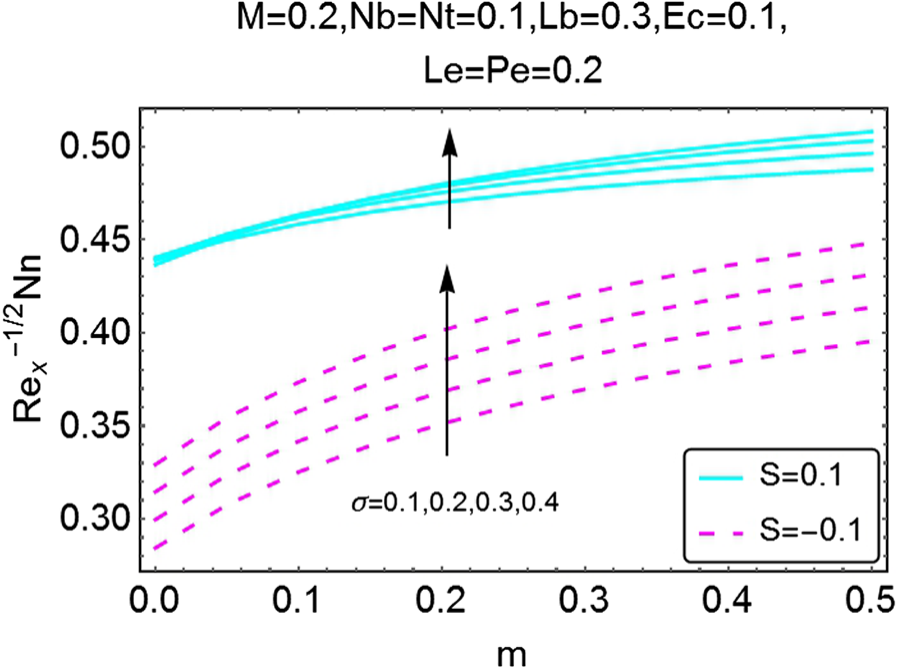
Variation in density number of motile microorganisms with *m* and *σ*

The variations in skin friction coefficient, Nusselt number and the density number of the motile microorganism, caused by the changes in different parameters, are plotted in Figs. 19, 20, 21, 22, 23, 24, 25, 26, and 27. From Fig. 19, an increment in the skin friction coefficient is observed for the increasing values of *m* and magnetic number *M*. A stronger magnetic field increases the skin friction coefficient. Here, the skin friction coefficient bears higher values in the case of injection as compared to suction.

A graphical description of the effects of *m* and magnetic number $M$ on Nusselt number is presented in Fig. 20. An interesting behavior is seen. With an increase in $m$, the value of Nusselt number decreases for the case of suction at the wall; while for the injection case, the same gets a rise. The influence of $M$ on Nusselt number is alike for both suction and injection cases, i.e. an increase in Nusselt number is observed. In Fig. 21, the influence of *Ec* on Nusselt number, due to the increasing values of $m$, is plotted. With an increase in $Ec$, there is a drop in the rate of heat transfer. Since *Ec* raises the temperature of the fluid, due to that the rate of heat transfer drops significantly. This behavior is same for both suction and the injection cases. Figures 22 and 23 give a description of effects of $m$, *Nb* and *Le* on Nusselt number. The Brownian motion decreases the temperature of the fluid, in a result, the rate of heat transfer at the wall increases (Fig. 22). An opposite behavior for the suction and the injection cases is also evident. Almost alike behavior of Nusselt number is observed for the increasing values of $Le$. For injection and suction at the wall, the rate of heat transfer is seen to be increasing with increasing values of $Le$. All these figures also show that the values of Nusselt number for the injection at the wall are on a higher side than the case of suction.

The next set of figures gives a description of the variations in density number of the motile microorganisms caused by the varying values of involved parameters. Figures 24, 25, 26, and 27 are plotted for the said purpose. The density number increases with increasing values of $m$ for both suction and the injection cases. For increasing *M*, the bioconvection Lewis number $Lb$, bioconvection number $Pe$ and the bioconvection constant *σ* give a rise in the density number of the motile microorganisms.

A comparison of the results obtained in this study with some already existing ones is tabulated in Table 1. It clearly shows that the solution obtained here is in an excellent agreement with the previous studies.

**Table 1:** Comparison of current results with already existing ones in the literature when $\varvec{ Pr} = 0.73$

*m*	$f^{{\prime \prime }} (0)$		$- \theta^{{\prime }} (0)$	
	Khan and Pop ( 2013)	Present	Khan and Pop (2013)	Present
0	0.4697	0.4690	0.4207	0.4201
0.0141	0.5047	0.5046	0.4263	0.4257
0.0435	0.5690	0.5689	0.4359	0.4354
0.0909	0.6550	0.6549	0.4477	0.4473
0.1429	0.7320	0.7320	0.4572	0.4569
0.2000	0.8021	0.8021	0.4653	0.4650
0.3333	0.9276	0.9276	0.4780	0.4781

## Conclusions

The flow and heat transfer of nanofluid in the presence of gyrotactic microorganisms is considered in a porous wedge. The MHD, Joule heating and viscous dissipation effects are also taken into consideration. Passive control model for the nanofluids is incorporated. The solutions are obtained numerically by using Runge–Kutta–Fehlberg method. The comparison of the solutions with some of already existing solutions is made which shows an excellent agreement between the solutions. A graphical analysis is carried out to analyze the behavior of velocity, temperature, concentration and the motile microorganisms density profiles. The major findings of this study are as under:

- The magnetic field increases the velocity of the fluid.
- Injection at the walls can be used to reduce the velocity boundary layer thickness.
- Thermal boundary layer thickness can be reduced by using the magnetic field and the suction at the wall.

The motile microorganisms density profile is an increasing function of the bioconvection Pecket number and bioconvection constant. The same is a decreasing function of *m*, *M* and *Le*.

The skin friction coefficient increases with increasing *m* and $M.$

- Nusselt number and the density number of motile microorganisms are higher for the case of suction as compared to the injection case.
- The density number of motile microorganisms is an increasing function for all the involved parameters.
